# Photoelectrochemical water oxidation over TiO_2_ nanotubes modified with MoS_2_ and g-C_3_N_4_

**DOI:** 10.3762/bjnano.13.127

**Published:** 2022-12-16

**Authors:** Phuong Hoang Nguyen, Thi Minh Cao, Tho Truong Nguyen, Hien Duy Tong, Viet Van Pham

**Affiliations:** 1 HUTECH University, 475A Dien Bien Phu Street, Binh Thanh District, Ho Chi Minh City, 700000, Vietnam; 2 Faculty of Engineering, Vietnamese-German University (VGU), Le Lai Street, Hoa Phu Ward, Thu Dau Mot City, Binh Duong Province, Vietnam

**Keywords:** band structure, g-C_3_N_4_/TiO_2_, MoS_2_/TiO_2_, photoelectrochemical, water splitting

## Abstract

TiO_2_ nanotube arrays (TNAs) have been studied for photoelectrochemical (PEC) water splitting. However, there are two major barriers of TNAs, including a low photo-response and the fast charge carrier recombination in TNAs, leading to poor photocatalytic efficiency. Through a comparison of MoS_2_/TNAs and g-C_3_N_4_/TNAs, it was found that TNAs modified with MoS_2_ and g-C_3_N_4_ exhibited a current density of, respectively, 210.6 and 139.6 μA·cm^−2^ at an overpotential of 1.23 V vs RHE, which is 18.2 and 12 times higher than that of pure TNAs under the same conditions. The stability of the MoS_2_/TNAs heterojunction is higher than that of g-C_3_N_4_/TNAs.

## Introduction

Hydrogen energy has become a target pursued in the energy development strategies of many countries and regions. Hydrogen is often synthesized via hydrocarbon compounds or water electrolysis [1]. Methods to produce hydrogen via electrochemical or photo-electrochemical (PEC) water splitting are considered a future direction of renewable fuel development [2–4]. The use of solar energy to activate catalytic materials to separate water for creating clean fuels has been developed for about a decade [5–6]. Water splitting is carried out in solutions rich in H^+^ ions to the conduct hydrogen evolution reaction (HER) process or in rich OH^−^ solutions for the oxygen evolution reaction (OER) process [7–9]. However, the electrode material must be extremely durable and nearly chemically inert to be able to withstand highly acidic or basic environments. Therefore, noble metals such as Pt, Pd, Au and Ag with suitable chemical properties, such as inertness, good resistance against corrosion and good electrical conductivity have been widely used in water splitting reactions [10–11]. However, noble metals are still rare and expensive materials, and their application as electrode materials is considered to be not optimal [10]. Therefore, the study of a materials with high-performance in PEC water splitting, which could replace noble metals are a research interest.

Photocatalytic technology uses semiconductors for effective approaches to the degradation of dyes and antibiotics, the removal of pollutant gases, and water splitting to produce hydrogen using solar energy [12–17]. Among such semiconductors, TiO_2_ nanotube arrays (TNAs) of 2–100 nm in diameter and 1–2 μm in length, are often used for efficient PEC applications exploiting advantages such as chemical stability, less toxicity and suitable cost [18–21]. However, there are two disadvantages affecting directly their photocatalytic ability. (i) TNAs only respond to ultraviolet (UV) light [22–24], and (ii) they exhibit fast carrier recombination [25]. Recently, the development of new heterojunction architectures through coupling TNAs with other semiconductor materials, especially low-bandgap semiconductors, led to a reduction of the required amounts of noble metals and materials such as CdS or ZnS [26–29]. There are many low-bandgap semiconductors that were coupled with TNAs, including MoS_2_, WS_2_, MoSe_2_, g-C_3_N_4_, Cu_2_O, and CuO. MoS_2_ is a semiconductor with a narrow bandgap (1.9 eV at room temperature) exhibiting unique chemical, thermal, and charge transport properties, which can shift the light absorption of TiO_2_ to the visible region [29–32]. An emerging new material in optoelectronics is g-C_3_N_4_ (bandgap of 2.65–2.7 eV) because it has an appropriate band structure with suitable energy levels regarding TiO_2_, which can improve the charge transfer states [33–34]. These two low-bandgap semiconductors improved considerably the PEC water splitting efficiency [35–36]. However, the fabrication of MoS_2_/TNAs and g-C_3_N_4_/TNAs has many disadvantages such as high synthesis temperatures, the requirement of a binder, or expensive synthesis equipment [29,36–38].

In this study, we compare properties and PEC water splitting efficiency of TNAs combined with the typical 2D materials MoS_2_ and g-C_3_N_4_ obtained with the same synthesis procedure. Insightful studies about optical and electronic properties have been conducted to explain clearly the difference between these composite materials

## Experimental

### Materials and chemicals

Chemicals and materials for the synthesis and characterization include Ti foil (1 cm × 2 cm), hydrochloric acid (HCl), sodium hydroxide (NaOH), DI water, acetone ((CH_3_)_2_CO), ethanol (C_2_H_5_OH), ammonium fluoride (NH_4_F), *N*-acetyl-ʟ-cysteine, ammonium heptamolybdate ((NH_4_)_6_Mo_7_O_24_), thiourea (CH_4_N_2_S), nitrogen gas, melamine, and nafion solution. All chemicals and materials were purified and used without further treatment.

### Preparation of materials

The individual materials including TNAs, MoS_2_, and g-C_3_N_4_ were synthesized as described earlier [39–41]. To combine with TNAs, 5 mg of MoS_2_ or g-C_3_N_4_ powder was dispersed in 2 mL of a solution containing 50 vol % ethanol and 50 vol % nafion solution as described in [40]. The solution was stirred for 30 min before ultrasonic treatment for 3 h to obtain a homogeneous solution. Next, 0.2 mL of either of these solutions was used to coat the surface of TNAs via spin coating. The samples were denoted as MoS_2_/TNAs and g-C_3_N_4_/TNAs. Then, the samples were annealed in nitrogen gas at 60 °C for 12 h to obtain a stable electrode for the investigation processes.

### Characterization of materials

The morphology, the phase, and the vibrational characteristics of the surface functional groups of the materials were observed by field-emission scanning electron microscopy (FESEM), X-ray diffraction (XRD), and Fourier-transform infrared spectroscopy (FTIR). Diffuse reflectance spectroscopy (DRS) was carried out to measure the optical bandgap of the semiconductor materials through the Tauc method using the absorption coefficient α of the material, according to Equation 1 [42]:


[1] $${\left(\alpha h\nu \right)}^{\frac{1}{\gamma }}=B\left(h\nu -E_{\text{g}}\right),$$

where *h*, ν, *E*_g_, and *B* are the Planck constant, the frequency of the photon, the bandgap energy, and a constant, respectively; γ = 1/2 for materials with direct bandgap and γ = 2 for semiconductor materials with an indirect bandgap.

### PEC activity evaluation

The PEC water splitting efficiency of the materials was evaluated through a three-electrode PEC cell using a Biologic SP-200 potentiostat. The three electrodes were a Pt counter electrode, a Ag/AgCl 3 M reference electrode, and a MoS_2_/TNAs or g-C_3_N_4_/TNAs working electrode in a 1 M Na_2_SO_4_ (pH 7.4) electrolyte solution. The light source used in this study was a 150 W Xe lamp (ABET Instruments) with a calibrated luminous intensity of 100 mW·cm^−2^ and a UV filter cutting at 380 nm.

## Results and Discussion

### Characterizations of materials

**Figure 1:**
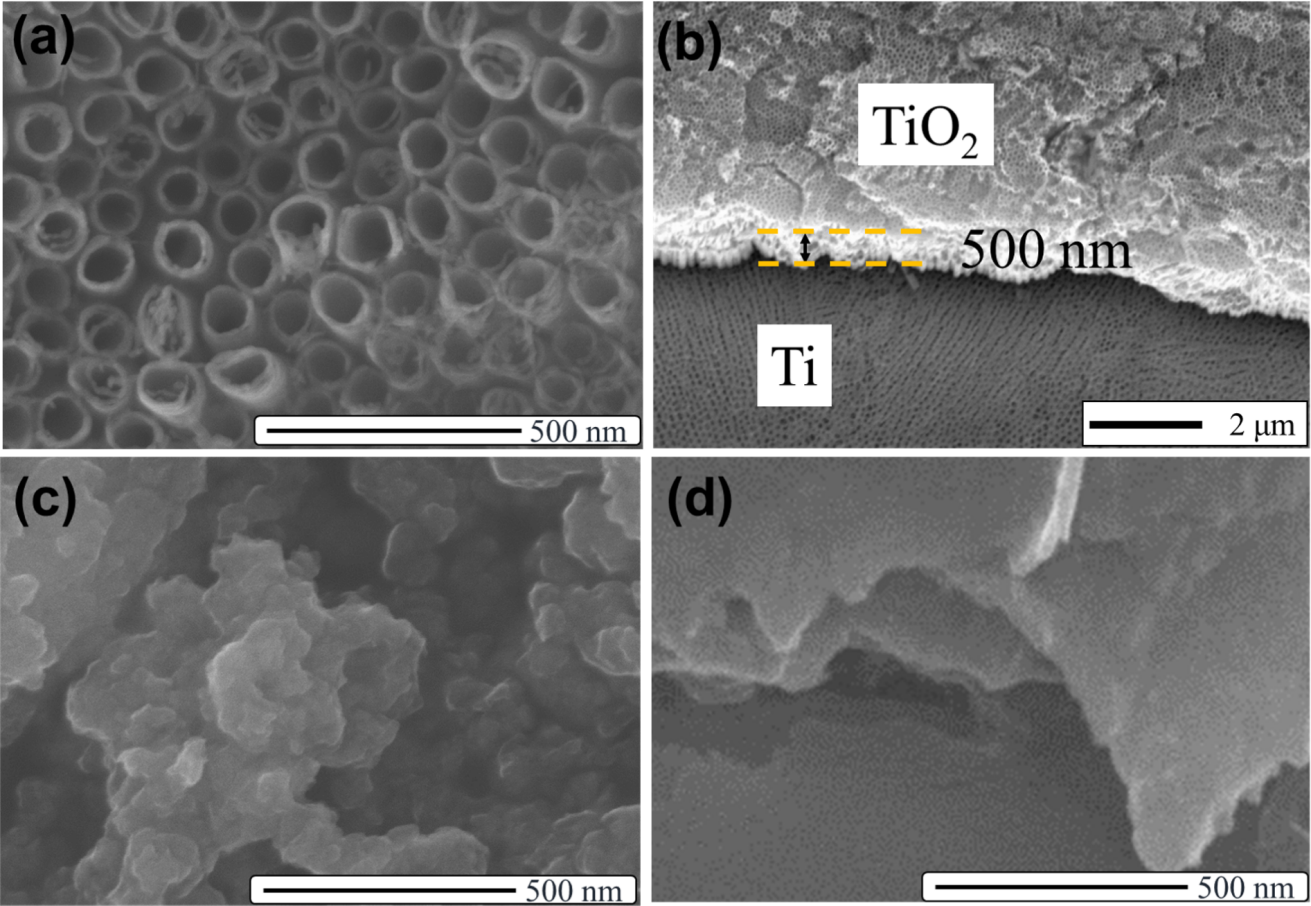
SEM images of TNAs (a, b), MoS_2_ (c), and g-C_3_N_4_ (d).

Figure 1a displays the morphology of TNAs, which have a uniform distribution of nanotubes with average diameters ranging from 80–100 nm and a length of 500 nm (Figure 1b). The MoS_2_ material exhibits the stacked layers of 2D materials (Figure 1b). This agrees with the results of previous publications in which hydrothermal methods were applied [24–26]. The SEM image of the g-C_3_N_4_ material shows the uniform nanosheets that were fabricated by the melamine pyrolysis method (Figure 1c).

**Figure 2:**
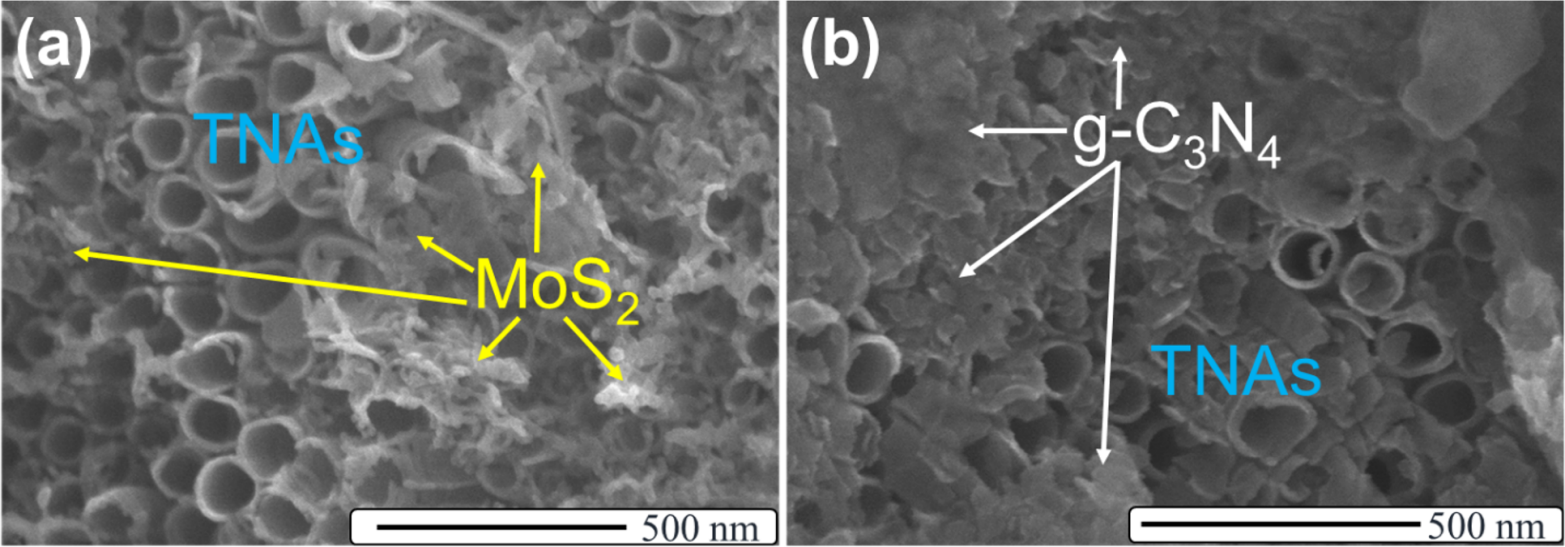
SEM images of MoS_2_/TNAs (a), and g-C_3_N_4_/TNAs (b).

After the deposition of 2D materials MoS_2_ and g-C_3_N_4_ onto the TNAs substrate, we examined the morphology of these heterostructures by using SEM (Figure 2). There are some small pieces that are randomly distributed on the surface of TNAs in Figure 2a, which were attributed to be MoS_2_. There is a similar result in the SEM image of g-C_3_N_4_/TNAs in Figure 2b. However, the distribution of g-C_3_N_4_ on the surface of the TNAs is denser than that of MoS_2_. Besides, the morphology of the TNAs did not change. The appearance of MoS_2_ and g-C_3_N_4_ has also been confirmed by EDS measurements and element mapping (Figure S1 and Figure S2, Supporting Information File 1).

**Figure 3:**
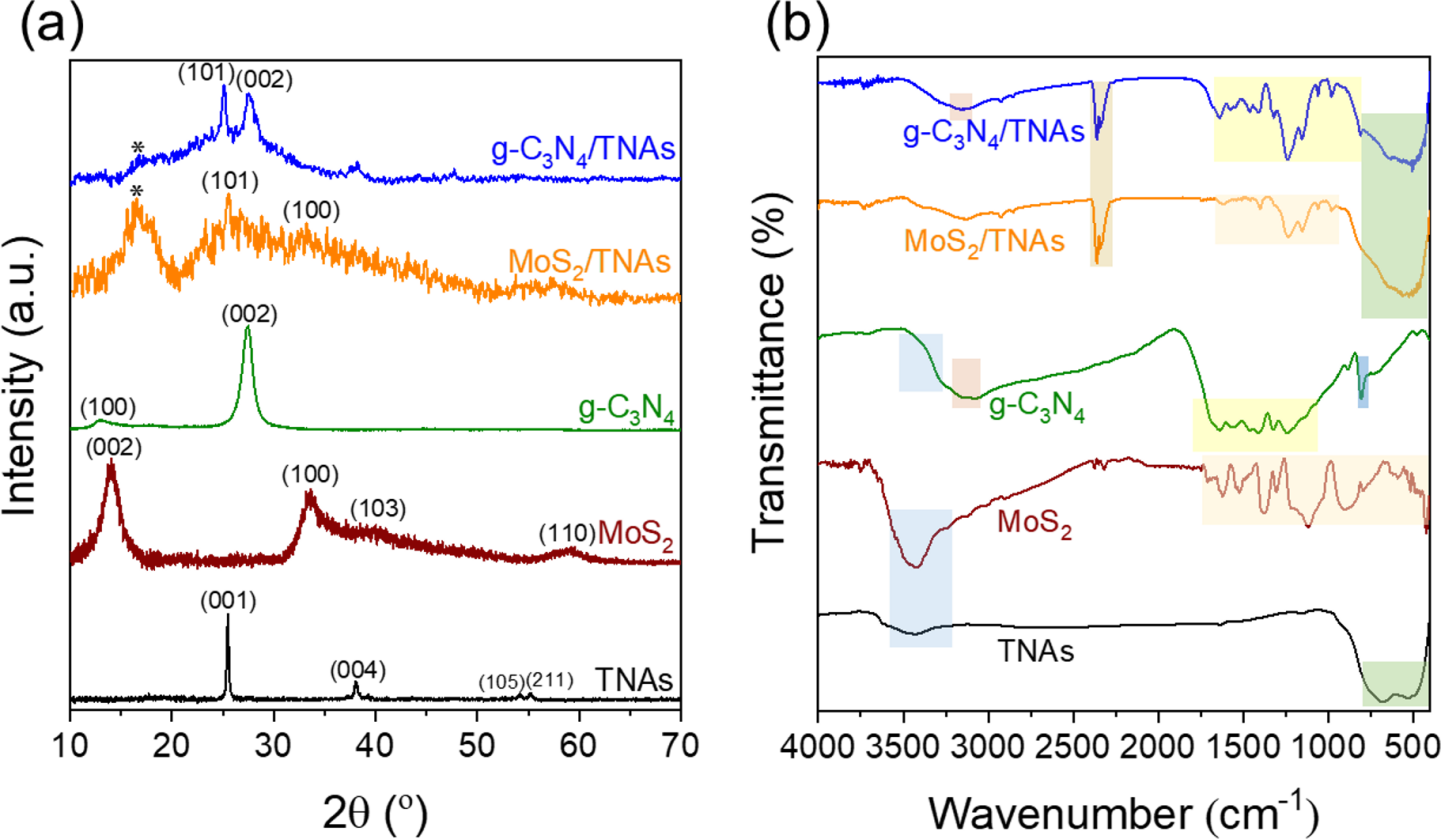
XRD pattern (a) and FTIR spectra (b) of as-synthesized samples.

Figure 3a shows that diffraction peaks of TNAs at 2θ = 25.45°, 37.07°, 39.24°, 54.10°, and 55.25°, attributed to the (101), (103), (004), (112), (105), and (211) planes of TiO_2_, respectively [JCPDS No. 21-1272]. Besides, the XRD pattern of MoS_2_ exhibits diffraction peaks at 13.97°, 33.56°, 40.24°, and 59.25°, corresponding to the (002), (100), (103), and (110) planes, respectively, of the 2H phase of MoS_2_ [JCPDS No. 37-1492]. The pristine g-C_3_N_4_ shows two distinct characteristic peaks at 2θ = 12.9° and 27.45°, assigned to the (100) and (002) planes, respectively [43–44]. The XRD diffraction results show the simultaneous appearance of diffraction peaks at 2θ = 25.45°, typical for the (101) planes of TNAs, and at 33.56° for the (001) planes of MoS_2_. Besides, the diffraction peak at 2θ = 16.45° characterizes the semi-crystalline structure of perfluorocarbon chains from nafion films [45]. Notably, the diffraction peak of the MoS_2_ material at 2θ = 13.97°, which is typical for the (002) lattice plane, is lost after the formation of the MoS_2_/TNAs heterostructure. This could be explained by the very small amount of MoS_2_ (5 mg) loaded onto the TNAs. Therefore, it will be difficult to identify the MoS_2_ phase in the composite from the XRD pattern of MoS_2_/TNAs. The (002) plane indicates the multilayer structure of MoS_2_ materials, the (001) plane indicates a monolayer structure of MoS_2_ [37,46]. Therefore, the disappearing (002) reflection and the remaining (001) reflection show that the ultrasonic treatment peeled the MoS_2_ material into thinner layered structures. This is in agreement with the SEM images, in which material with rather small and thinner structures scattered on the surface of TNAs was observed.

The functional groups and chemical bonds of the as-prepared materials were determined by using FTIR spectroscopy, as shown in Figure 3b. The formation of TiO_2_ on the Ti foil is indicated by the vibrations of the Ti–O bond in the wavenumber region from 450 to 750 cm^−1^ [47]. The bonding characteristics in the MoS_2_ material are presented by Mo–S vibration peaks between 1620 and 420 cm^−1^ [48]. Also, FTIR spectroscopy is used as an extremely effective technique for studying the vibrational states of organic bonds in g-C_3_N_4_ materials using the vibrational peaks of C–N bonds between 1640 and 1200 cm^−1^. A very strong absorption peak at 807.2 cm^−1^ characterizes the fluctuation of tri-s-triazine of g-C_3_N_4_. Vibrational peaks in the 3200 cm^−1^ region attributed to fluctuations of the C–N group also appeared [49]. Figure 3b shows the bonding states in the MoS_2_/TNAs and g-C_3_N_4_/TNAs heterostructures. The results show that, in addition to the typical bonding of the TNAs substrate such as Ti–O bonds, there are vibrations of composites of TNAs with MoS_2_ (between 420 and 1620 cm^−1^) and g-C_3_N_4_ (between 1200 and 1640 cm^−1^ for C–N bonds and 807 cm^−1^ for tri-s-triazine subunit). The peaks in the wavenumber range between 3400 and 1625 cm^−1^ of all samples are typical for stretching vibrations of the O–H bonds, which are caused by air humidity.

**Figure 4:**
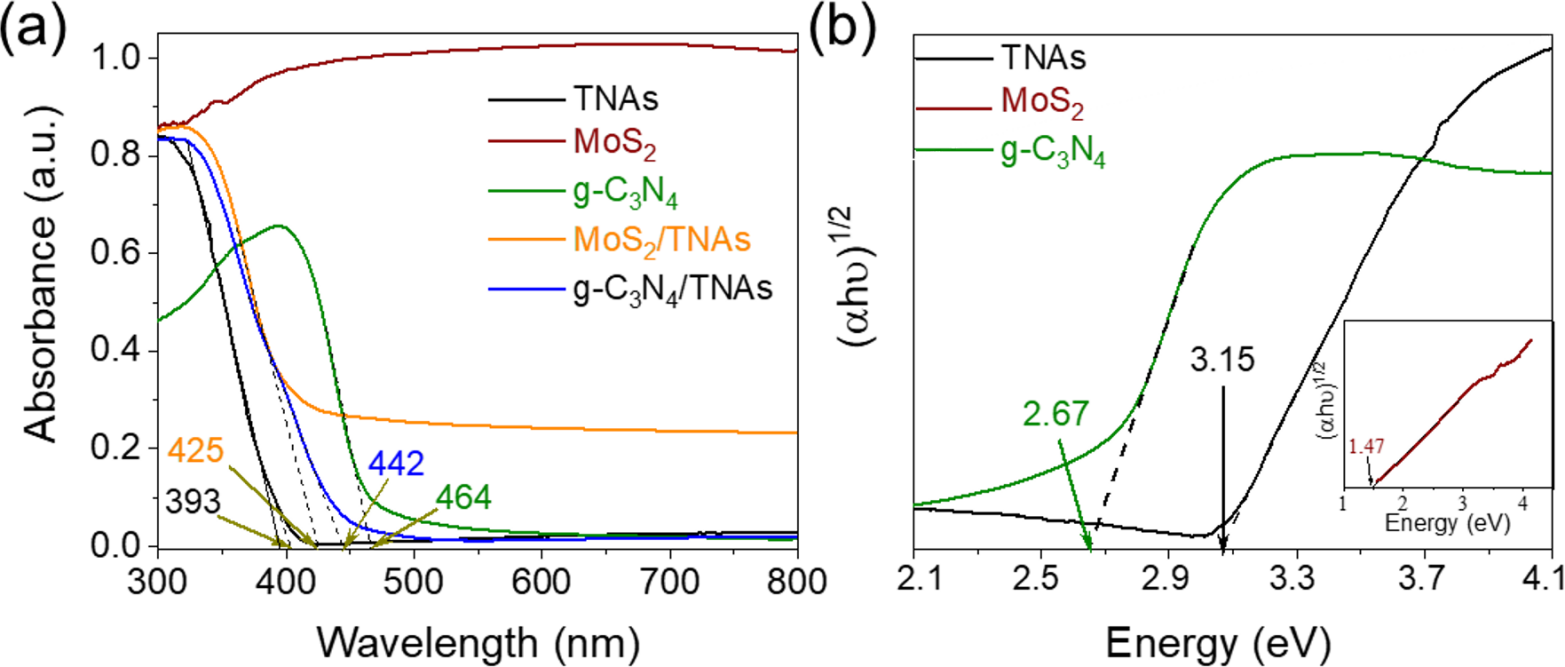
Comparison of the optical properties of as-synthesized materials through DRS spectra (a) and Tauc plots (b).

To confirm the ability of the heterojunctions to enhance absorption in the visible-light region, the DRS spectra and Tauc plots were recorded and are presented in Figure 4. It can be easily observed in Figure 4a that the TNAs show a strong absorption edge at 393 nm. This means that TNAs are only activated by near-UV irradiation. In contrast, the g-C_3_N_4_ sample shows an absorption edge at 464 nm. Meanwhile, MoS_2_ exhibits strong absorption from the UV region extending to the entire visible-light region. It can be seen that the loading of both MoS_2_ and g-C_3_N_4_ on the TNAs surface extended the absorption to the visible-light range. Specifically, the absorption edge of the g-C_3_N_4_/TNAs and MoS_2_/TNAs samples shifted to 442 and 425 nm, respectively. Besides, a remarkable feature of the DRS spectrum of MoS_2_ is a superior absorption ability in the whole visible-light range in comparison to that of the remaining samples. To evaluate the optical bandgap energy of TNAs and g-C_3_N_4_, Tauc plots were extrapolated in Figure 4b. The bandgap values of TNAs, g-C_3_N_4_, and MoS_2_ were calculated as about 3.15, 2.67, and 1.47 eV, respectively. These results are agreement with previous publications [50–52].

**Figure 5:**
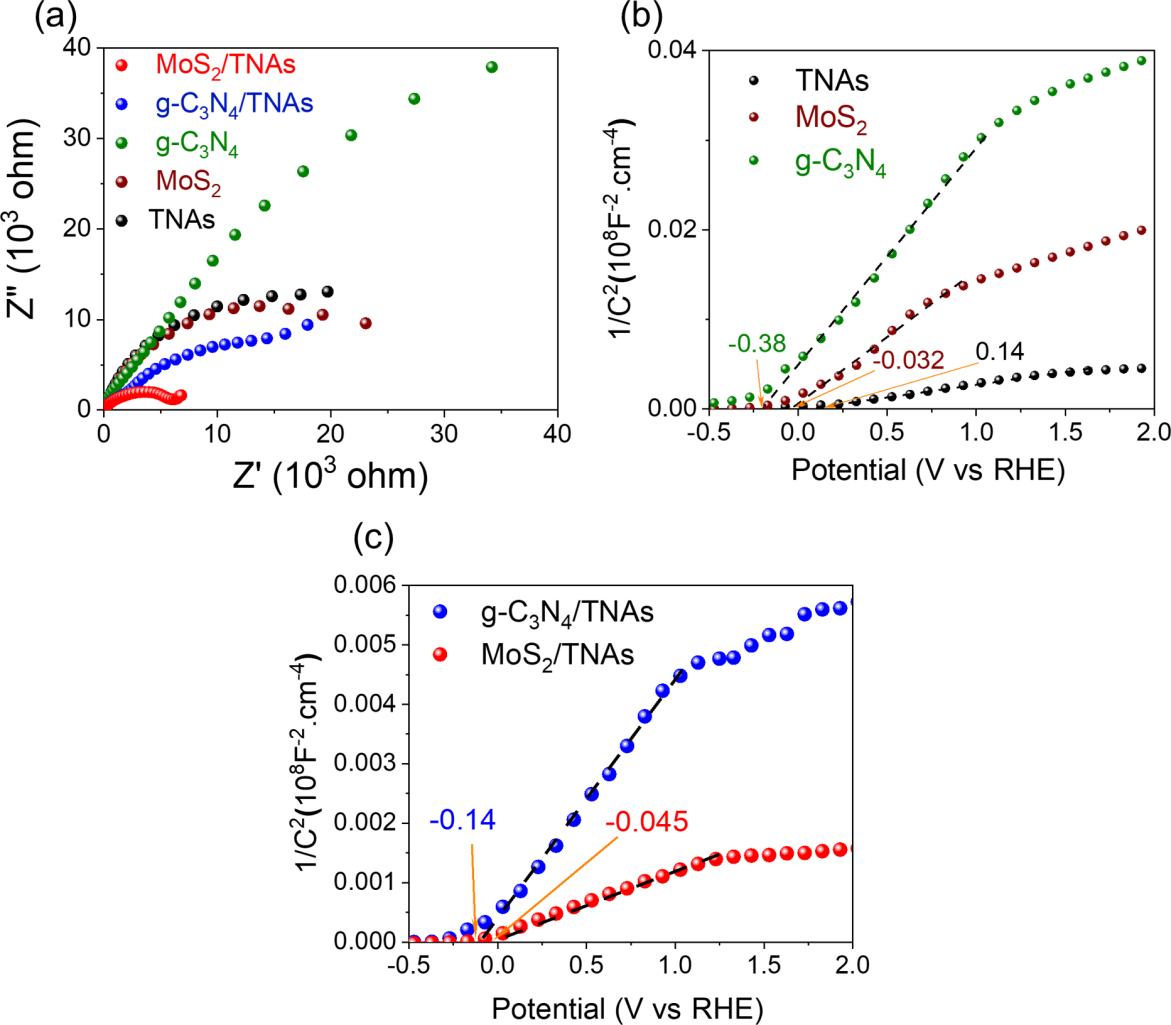
EIS spectra (a), Mott–Schottky plots of pristine materials (b) and heterostructures (c).

Figure 5 shows the results of electrochemical impedance spectroscopy (EIS), that is, Nyquist and Mott–Schottky plots of the materials, which give information about the charge transfer mechanism at the interface. In Figure 5a, the Nyquist plots of the samples all exhibit only single semicircular shape, which shows the charge transfer resistance equivalent to the polarization resistance. This result also demonstrates a unique interaction of the electrode surface and the electrolyte solution. Furthermore, the g-C_3_N_4_ sample shows the semicircle with the largest radius, followed by TNAs and MoS_2_, which indicates the low interaction of these materials with the electrolyte. However, after coupling, the g-C_3_N_4_/TNAs sample shows a semicircle with smaller radius compared than that of g-C_3_N_4_ or TNAs. The Nyquist plot of the MoS_2_/TNAs sample shows the smallest semicircle radius of all samples. This result indicates an increase in carrier density during the reaction with the electrolyte solution, leading to a decrease in resistance of the g-C_3_N_4_/TNAs and MoS_2_/TNAs samples. This could be explained by the enhanced optical interaction, causing an increase of the carrier concentration in MoS_2_/TNAs sample such in Figure 4.

The mechanism for the enhanced activity of the heterojunctions can be explained by the Mott–Schottky results in Figure 5b,c. Generally, all samples show positive slopes, which proves that they are n-type semiconductors [53]. Equation 2 shows the Mott–Schottky relationship involving the apparent capacitance as a function of the potential under depletion conditions [54]:


[2] $$C^{-2}=\frac{2}{e\varepsilon \varepsilon_{0}N_{d}A^{2}}\left(V_{\text{a}}-V_{\text{fb}}-\frac{kT}{e}\right),$$

where *C*, ε, ε_0_, *N*, *A*, *V*_a_, *V*_fb_, *k*, and *T* are the capacitance of the space charge region, the dielectric constant of the semiconductor, the vacuum permittivity, the donor density, the area of interface or the electrode, the applied and flat band potentials, the Boltzmann constant, and the temperature, respectively.

The plot of 1/*C*^2^ vs *V* shows an intercept of the x-axis, which corresponds to the flat band potential (*E*_fb_), that is, the conduction band maximum (CBM) level of the material. The Mott–Schottky plots of TNAs, g-C_3_N_4_, and MoS_2_ samples yield *E*_fb_ (or CBM) values of 0.14, −0.38, and −0.032 V vs RHE, respectively. It can be found that the *E*_fb_ values of g-C_3_N_4_ and MoS_2_ are significantly more negative than that of TNAs, which can facilitate the migration of electrons from g-C_3_N_4_ and MoS_2_ to TNAs. Furthermore, the *E*_fb_ values of g-C_3_N_4_/TNAs and MoS_2_/TNAs are shifted to −0.14 and −0.045 V vs RHE (Figure 5c). The heterostructures express much more negative *E*_fb_ values than pristine TNAs, which is attributed to the enhanced electron density, the depletion of the *E*_fb_, and electron–hole recombination [55].

### PEC characterizations of materials

**Figure 6:**
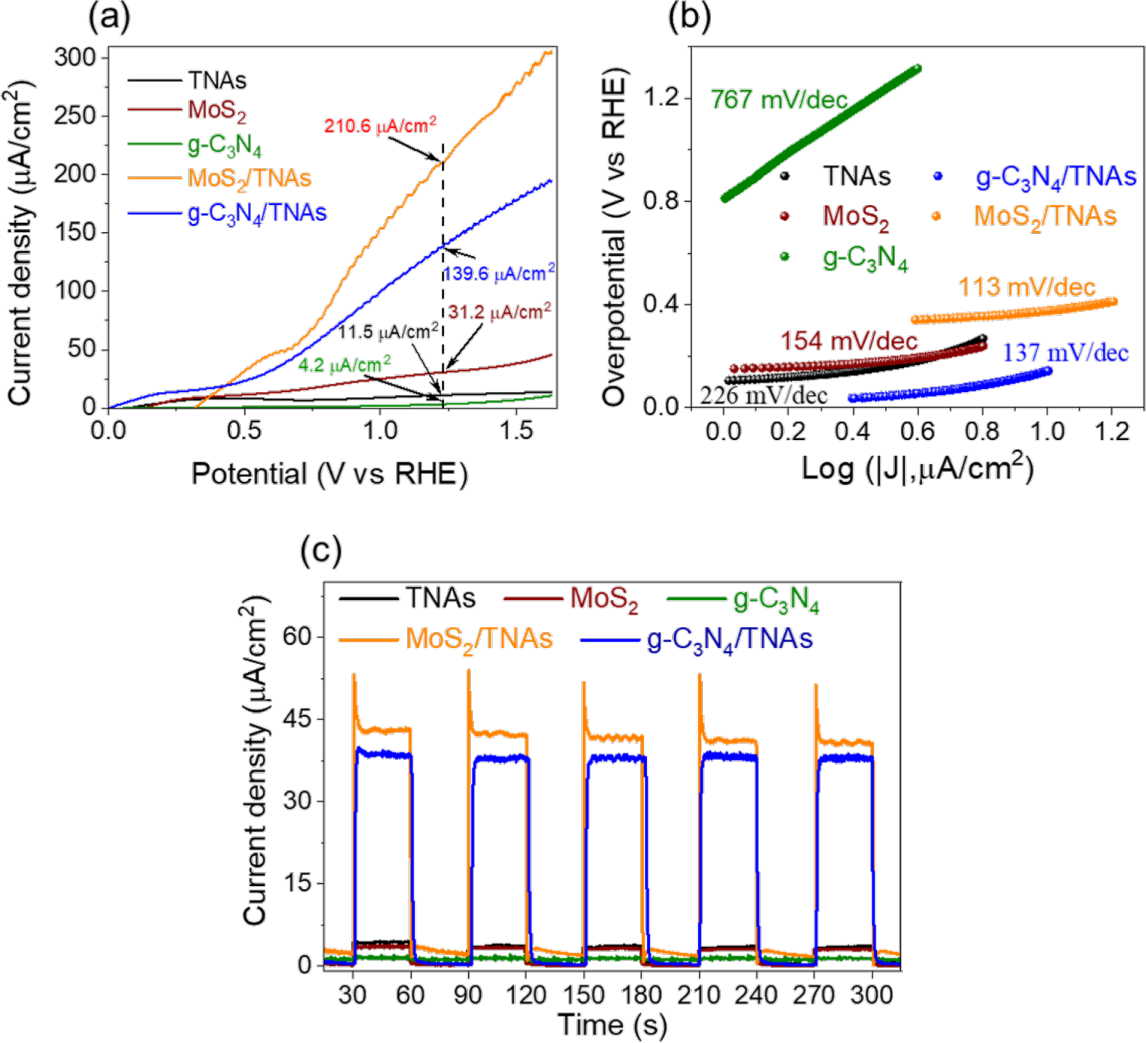
LSV plots (a), Tafel slopes (b), and photo-response (c) of the materials.

Figure 6 shows the linear sweep voltammetry (LSV) curves, Tafel slopes, and the photo-response of the samples. Figure 6a shows that the current density of all materials is grows linearly with the applied potential under visible-light excitation. The onset potentials of the of TNAs, g-C_3_N_4_, and MoS_2_ are 0.08, 0.16, and 0.14 V vs RHE, respectively. Further, for the OER (1.23 V vs RHE), the current densities of TNAs, g-C_3_N_4_, and MoS_2_ are 11.5, 4.2, and 31.2 µA/cm^2^, respectively. The onset potential values of g-C_3_N_4_/TNAs and MoS_2_/TNAs are significantly shifted to −0.76 and 0.008 V, respectively. In addition, the current density also exhibited an improvement with values of 139.6 and 210.6 µA/cm^2^ at 1.23 V for g-C_3_N_4_/TNAs and MoS_2_/TNAs, respectively, which shows their superiority in the PEC water oxidation reaction. The LSV results are also consistent with the previous results from EIS analysis and the Mott–Schottky results (Figure 5). The PEC activity of MoS_2_/TNAs in this study is higher than that of MoS_2_/TNAs synthesized by using a PVA binder agent in [36]. However, the direct combination of g-C_3_N_4_ with TNAs at a relatively high fabrication temperature (500 °C for 2 h) in [35] yielded better results better than those of this study. The investigation of the stability of the PEC electrodes from MoS_2_/TNAs and g-C_3_N_4_/TNAs is described in Figure S3, Supporting Information File 1. After every PEC test cycle, we immersed the PEC electrode in DI water for 1 h and let it dry completely before the next test. We can conclude that the stability of the MoS_2_/TNAs heterojunction is higher than that of the g-C_3_N_4_/TNAs heterojunction. The decrease in catalytic activity of the PEC electrodes is explained by the leaching of the catalysts MoS_2_ and g-C_3_N_4_ after each activity measurement.

The Tafel slope is considered as an important parameter to evaluate the PEC activity in water splitting. A smaller Tafel slope value indicates a more rapid increase in the reaction rate of the electrode. Figure 6b shows the high Tafel slope values of the individual materials, TNAs, g-C_3_N_4_, and MoS_2_, of 226, 767, and 154 mV/dec, respectively. After the modification, the Tafel slope value of g-C_3_N_4_/TNAs is only about 137 mV/dec, while the best Tafel slope value of MoS_2_/TNAs is only 113 mV/dec. Furthermore, the photocurrent of the materials was evaluated through the assessment of the photo-response under visible-light irradiation at 0.63 V in Figure 6c. A current density of about 38.6 µA/cm^2^ was obtained with the g-C_3_N_4_/TNAs even after five cycles, which is nearly ten times higher than that of pure TNAs. The current density of MoS_2_/TNAs is even higher than that of g-C_3_N_4_/TNAs reaching 43.4 µA/cm^2^ after five cycles. These results indicate that enhancement of the optical interaction in MoS_2_/TNAs heterostructures is stronger than that in g-C_3_N_4_/TNAs [56–57]. Further, the current density increases sharply and decreases rapidly within a few seconds for MoS_2_/TNAs under light, which can be explained as follows: The photocurrent density of MoS_2_/TNAs promptly increased because of the efficient separation of the e^−^–h^+^ pairs at the interfaces between TNAs and MoS_2_ [58] and the rapid transfer of the photo-induced electrons from MoS_2_ to the TNAs electrode [59]. This result is in agreement with the EIS results in Figure 5a, where the arc radius of the Nyquist plot of MoS_2_/TNAs was the smallest, indicating that MoS_2_/TNAs effectively decreased the resistance of the TNAs and, thus, speeded up the charge transfer on the photoelectrode. These arguments are consistent with results previously published in [58].

**Figure 7:**
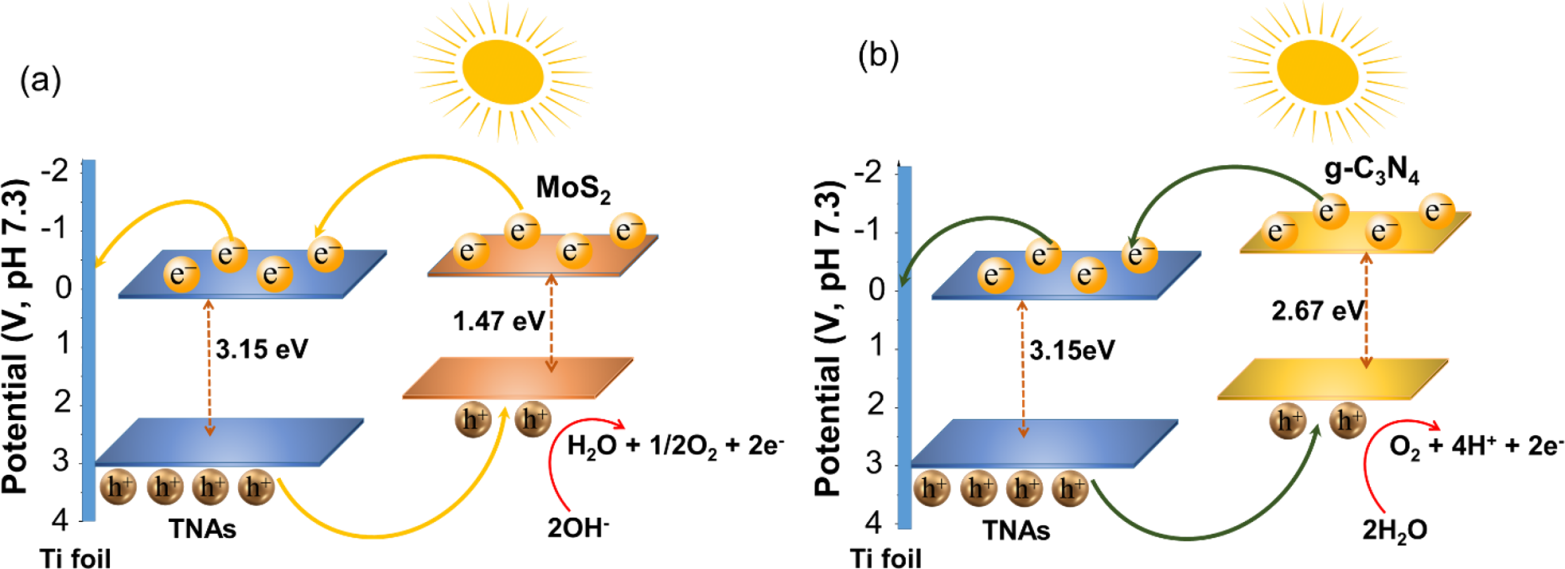
Proposed band diagram of MoS_2_/TNAs (a) and g-C_3_N_4_/TNAs (b).

Figure 7 presents the energy band diagram structure of the MoS_2_/TNAs and g-C_3_N_4_/TNAs heterojunctions based on the DRS and Mott–Schottky analysis results, which are summarized in Table 1.

**Table 1:** The CBM and *E*_g_ values of the materials.

Sample	CBM level (V vs RHE, pH 7.3)	*E*_g_ (eV)

TNAs	0.14	2.99
MoS_2_	−0.032	1.47
g-C_3_N_4_	−0.38	2.63

It is easily observed from Figure 7 that the heterostructures formed upon incorporation of TNAs with MoS_2_ and g-C_3_N_4_ are all of type II. Type-II heterostructures promote the migration of h^+^ and e^−^ under visible-light irradiation. Electrons can move from the conduction band (CB) of MoS_2_ or g-C_3_N_4_ to the CB of TNAs in MoS_2_/TNAs or g-C_3_N_4_/TNAs, respectively. In contrast, holes will migrate from the valence band (VB) of TNAs to the VB of MoS_2_ or g-C_3_N_4_. Therefore, the recombination of the photo-generated e^−^–h^+^ pairs is reduced. In this contribution, the PEC water splitting reactions take place in a neutral media, which is well known to occur via two processes, including the oxidation and reduction reactions at, respectively, the anode and cathode described by Equation 3 and Equation 4.

Oxidation reaction at the anode:


[3] $$2{\text{OH}}^{-}+2{\text{h}}_{\text{VB}}^{+}\to 2{\text{H}}^{+}+{\text{O}}_{2}+2{\text{e}}^{-}.$$

Reduction reaction at the cathode:


[4] $$2{\text{H}}^{+}+2{\text{e}}^{-}\to {\text{H}}_{2}.$$

Carrying out the reactions in a neutral medium also contributes to the increased durability of the electrodes. However, the lack of initial H^+^ concentration can reduce the efficiency of the H_2_ production. For an effective water splitting process, the oxidation reaction of OH^−^ ions in the electrolyte needs to take place at the anode to generate e^−^ and H^+^ ions along with O_2_. The e^−^ current will immediately migrate to the cathode to perform reduction reactions. At that time, H^+^ will also be reduced at the cathode to form H_2_. The higher the efficiency of the oxidation reaction, the more e^−^ are generated and the stronger the H^+^ reduction reaction will be. Preventing recombination of photo-generated e^−^–h^+^ pairs in the MoS_2_/TNAs and g-C_3_N_4_/TNAs heterojunction structures has also been shown to increase the efficiency of PEC water splitting.

## Conclusion

MoS_2_/TNAs and g-C_3_N_4_/TNAs heterojunctions have been successfully fabricated for PEC water splitting. The role of g-C_3_N_4_ and MoS_2_ in mitigating the recombination of e^−^–h^+^ pairs has been demonstrated. The ability to enhance the optical interaction of the heterostructures was presented through the reduction of the bandgap energy. The outstanding application performance of g-C_3_N_4_/TNAs and MoS_2_/TNAs combinations was presented. In detail, the excellent water-splitting ability of g-C_3_N_4_/TNAs and MoS_2_/TNAs heterojunctions achieved about 139.6 and 210.6 µA/cm^2^. In addition, the PEC reaction rate was evaluated by the Tafel slope value, indicating a faster rate for the MoS_2_/TNAs heterojunction compared to the g-C_3_N_4_/TNAs heterojunction. Moreover, the photocurrent density of MoS_2_/TNAs is higher than that of g-C_3_N_4_/TNAs due to the strong optical interaction of the MoS_2_/TNAs heterostructure.

## Supporting Information

File 1 Additional figures.
