# Supplementary material for: Photoelectrochemical water oxidation over TiO2 nanotubes modified with MoS2 and g-C3N4
Source: Beilstein J Nanotechnol. 2022 Dec 16;13:1541–50. doi: 10.3762/bjnano.13.127 (PMC9764855; doi:10.3762/bjnano.13.127)
Supplement: File 1 — Additional figures. [file Beilstein_J_Nanotechnol-13-1541-s001.pdf]

BEILSTEIN JOURNAL OF NANOTECHNOLOGY

Supporting Information

for

Photoelectrochemical water oxidation over TiO_2 nanotubes modified with MoS_2 and g-C_3N_4

Phuong Hoang Nguyen, Thi Minh Cao, Tho Truong Nguyen, Hien Duy Tong and Viet Van Pham

Beilstein J. Nanotechnol. **2022,** *13,* 1541–1550. doi:10.3762/bjnano.13.127

Additional figures

License and Terms: This is a supporting information file under the terms of the Creative Commons Attribution License (https://creativecommons.org/licenses/by/4.0). Please note that the reuse, redistribution and reproduction in particular requires that the author(s) and source are credited and that individual graphics may be subject to special legal provisions.
The license is subject to the *Beilstein Journal of Nanotechnology* terms and conditions: (https://www.beilstein-journals.org/bjnano/terms)

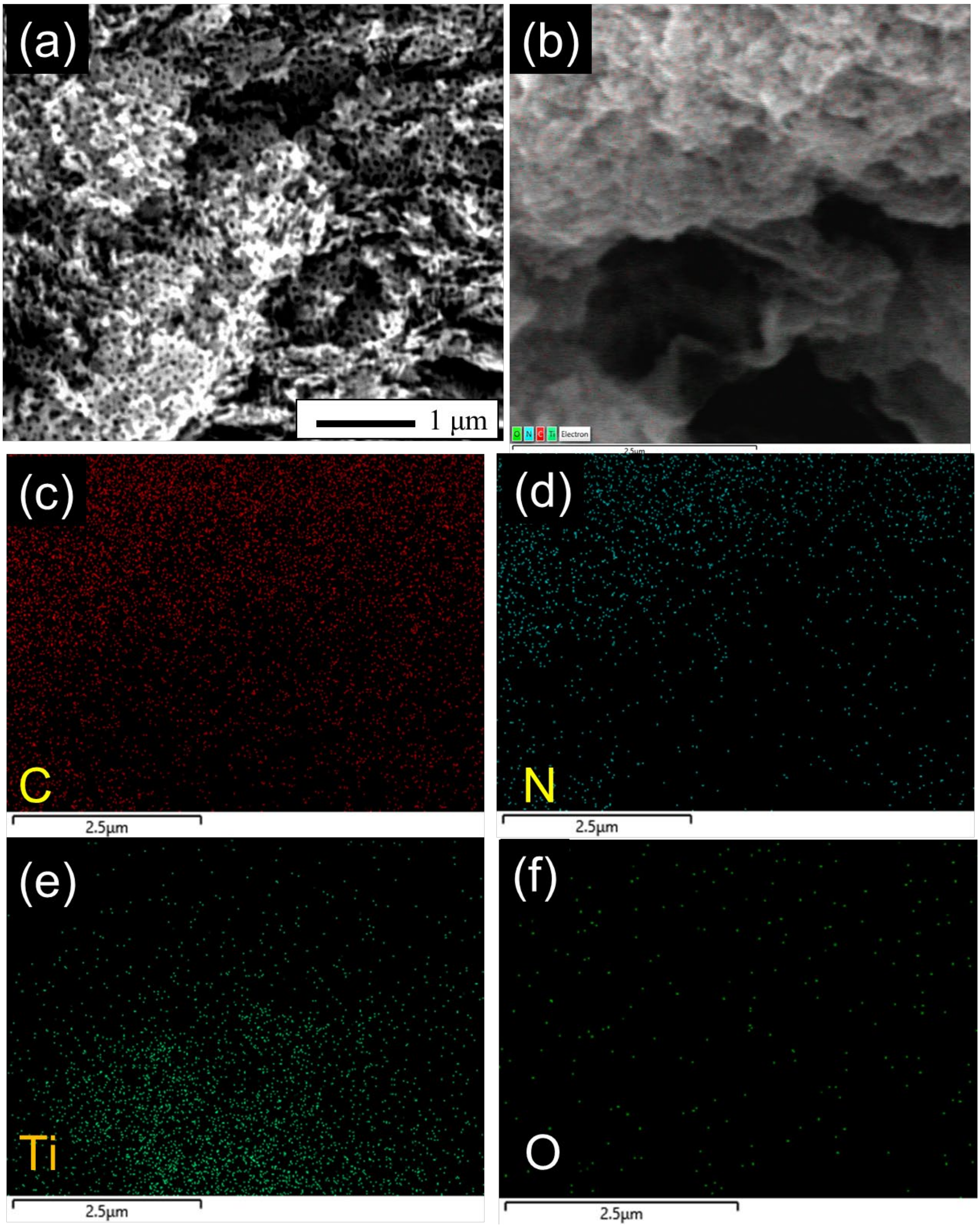

Figure S1: Characterization of g-C_3N_4/TNAs materials: (a) SEM image, (b) EDS layered image, and (c–f) element mapping.

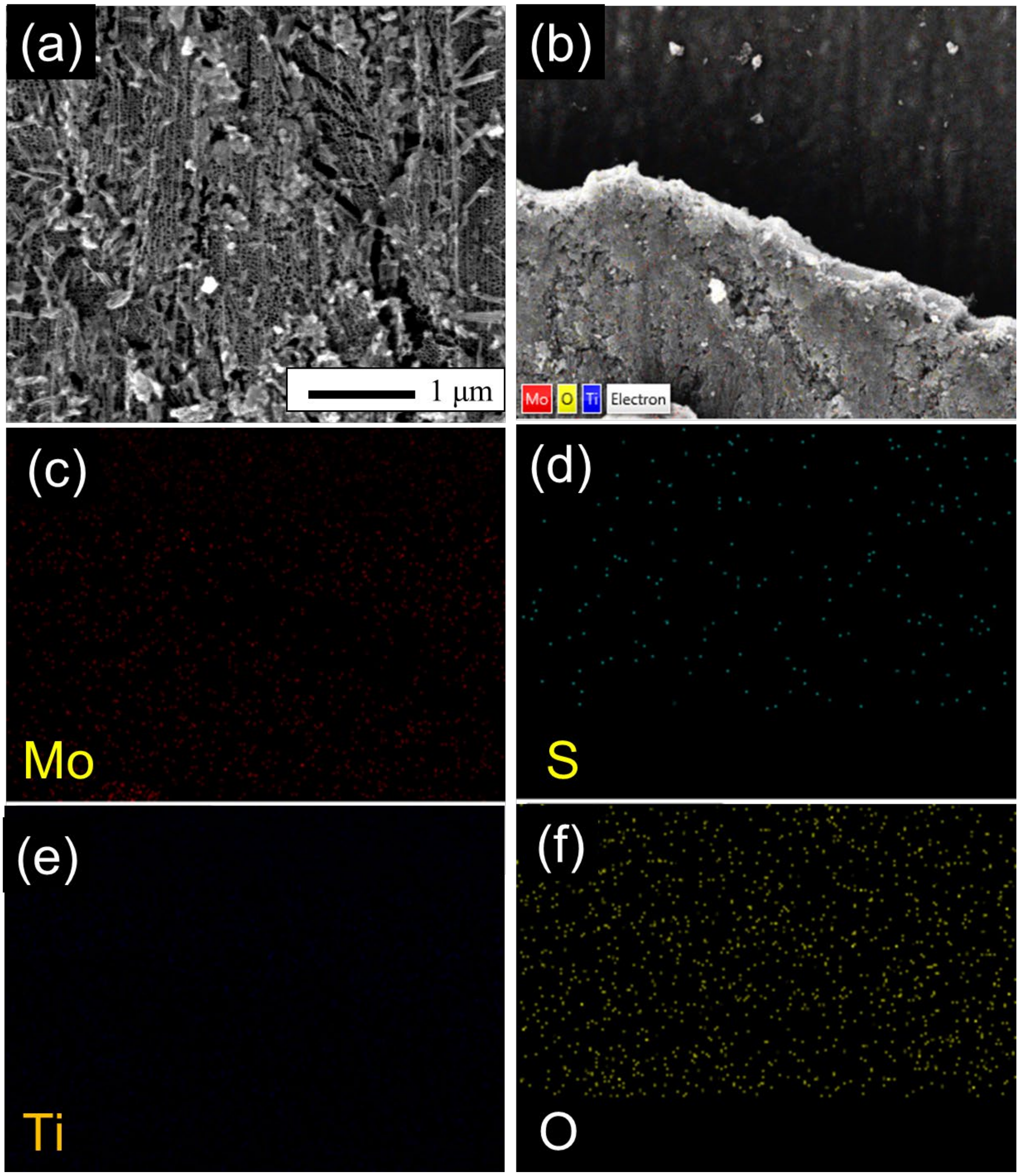

Figure S2: Characterization of MoS_2/TNAs materials: (a) SEM image, (b) EDS layered image, (c–f) and element mapping.

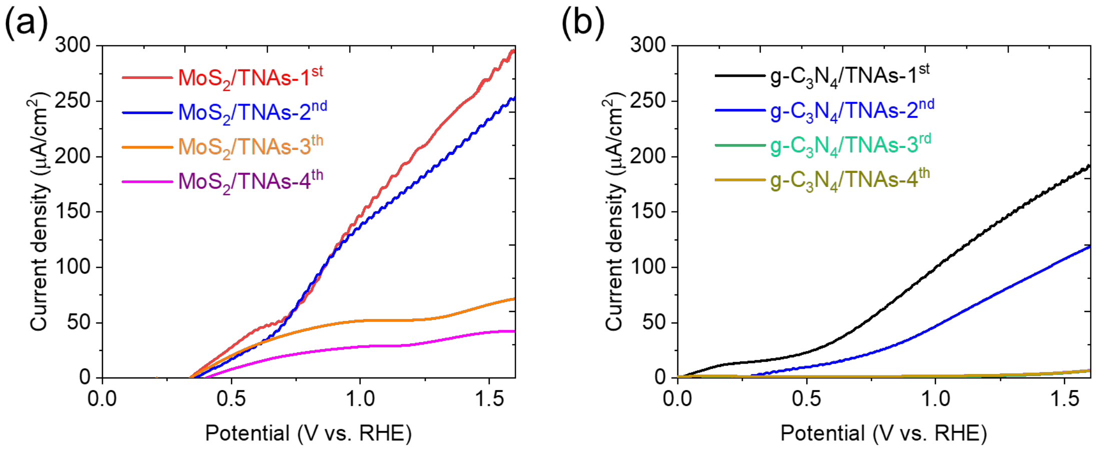

Figure S3: Stability of the MoS_2/TNAs and g-C_3N_4/TNAs heterojunction. After every PEC test cycle, the PEC electrode is immersed in DI water for 1 h and after that it is dried completely before the next test.
